# Microhardness and Tensile Strength Analysis of SS316L/CuCrZr Interface by Laser Powder Bed Fusion

**DOI:** 10.3390/ma17122836

**Published:** 2024-06-11

**Authors:** Xiang Jin, Zhiong Sheng Hoo, Chuanjie Jin, Zhongmin Xiao, Liming Yao

**Affiliations:** 1 School of Mechanical Science and Engineering, Northeast Petroleum University, Daqing 163318, China; 13653458637@163.com (X.J.); jinchuanjie666@163.com (C.J.); 2 School of Mechanical and Aerospace Engineering, Nanyang Technological University, 50 Nanyang Avenue, Singapore 639798, Singapore; zhiongsh001@e.ntu.edu.sg; 3 School of Mechatronics Engineering, Harbin Institute of Technology, Harbin 150001, China; 4 Zhengzhou Research Institute, Harbin Institute of Technology, Zhengzhou 450000, China

**Keywords:** additive manufacturing, L-PBF, dissimilar metal, microhardness, tensile strength

## Abstract

Metallic joints within tokamak devices necessitate high interface hardness and superior bonding properties. However, conventional manufacturing techniques, specifically the hot isostatic pressing (HIP) diffusion joining process, encounter challenges, including the degradation of the SS316L/CuCrZr interface and CuCrZr hardness. To address this, we explore the potential of laser powder bed fusion (LPBF) technology. To assess its viability, we fabricated 54 SS316L/CuCrZr samples and systematically investigated the impact of varied process parameters on the microhardness and tensile strength of the dissimilar metal interfaces. Through comprehensive analysis, integrating scanning electron microscopy (SEM) imagery, we elucidated the mechanisms underlying mechanical property alterations. Notably, within a laser volumetric energy density range of 60 J/mm^3^ to 90 J/mm^3^, we achieved elevated interface hardness (around 150 HV) and commendable bonding quality. Comparative analysis against traditional methods revealed a substantial enhancement of 30% to 40% in interface hardness with additive manufacturing, effectively mitigating CuCrZr hardness degradation.

## 1. Introduction

**Figure 1:**
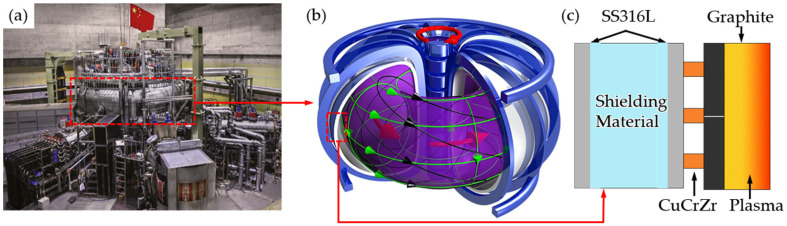
(**a**) Tokamak device in the nuclear industry; (**b**) schematic of Tokamak device structure; (**c**) cross-section of a vacuum radiation shielding structure [2].

The tokamak device [Figure 1a,b] is a toroidal vessel based on the magnetic confinement principle, which is employed to realize controlled nuclear fusion [1]. The vacuum radiation shielding structure is of high research value as the core component of heat insulation and radiation protection. A typical cross-section of a vacuum radiation shielding structure is shown in Figure 1c [2]. In this structure, CuCrZr serves the function of heat dissipation, while a double-wall configuration comprising an SS316L plate and an internal radiation shielding material is employed to shield fusion neutrons. The heat flux of the radiation shielding structure is 1–10 MW/m^2^, indicating that the SS316L/CuCrZr interface requires a high degree of bonding quality and is typically connected by the hot isostatic pressing (HIP) diffusion joining process in practice [3]. The fundamental principle of HIP diffusion joining technology is to induce solid-phase diffusion of atoms at the interface of the joining through the action of high temperature and high pressure [4,5]. The interface exhibits high strength, microstructural integrity, and low distortion. Nevertheless, it is not the optimal manufacturing process for producing dissimilar metal joints [6,7]. From the perspective of the joining process, HIP diffusion joining technology necessitates a high surface quality of the connection interface, which must be connected after complex pretreatment, requiring hours or even days of joining time. Furthermore, the precise control of temperature and contact pressure is essential during the joining process [8,9]. From a practical production standpoint, the interface of SS316L/CuCrZr structures produced by HIP diffusion joining technology, as well as CuCrZr, is susceptible to hardness degradation. In a recent study, Singh, K. P. et al. produced SS316L/CuCrZr joints using HIP diffusion joining technology and investigated the effect of different process parameters on the interfacial microhardness with the assistance of Ni intercalation [10]. The interfacial region hardness ranged from 52.4 HV to 112.2 HV, while the CuCrZr hardness ranged from 63.4 HV to 84.5 HV. These values were much lower than the pure CuCrZr hardness (110 HV). The team also verified the feasibility of using vacuum brazing technology to produce joints [11]. Although this technique has a smaller heat-affected zone and is less prone to stress concentrations than HIP diffusion joining and conventional welding techniques, it is observed that the hardness degradation is more pronounced.

Additive Manufacturing (AM) technology, as a novel manufacturing method, is distinguished by its rapid molding speed, high manufacturing precision, and excellent mechanical properties [12,13,14]. Currently, it is employed in a number of fields, including aerospace [15,16], automotive [17,18], biomedical [19,20], and others. In the field of nuclear engineering, numerous industry norms and standards prohibit the utilization of AM components. Consequently, a number of pressurized structure AM components, including miniature heat exchangers [21,22], storage tanks [23,24], and pump units [25], remain in the experimental phase. Nevertheless, in non-pressurized structures, AM technology has made significant advancements, with a particular focus on the production of fuel, cladding, and control components within the reactor core [26,27,28]. Westinghouse was one of the first organizations to propose the installation of AM assemblies in reactors. The thimble clogging device produced by the company is currently undergoing field trials. Furthermore, AM components [High Flux Isotope Reactor (HFIR) and AM Capsule] at Oak Ridge and Idaho National Laboratory have entered the trial stage [29,30], and AM components are also used in spent fuel recycling and reuse [31]. The majority of the aforementioned outcomes are based on the advantages of AM technology in forming complex structures. However, there has been no reported application of multi-metallic AM technology in the nuclear industry. In comparison to the HIP diffusion joining technology, the production of SS316L/CuCrZr joints by AM technology offers a significant advantage, particularly at the interface. This can be achieved without the need for complex surface treatment on both sides of the joints and subsequent HIP diffusion joining, which greatly simplifies the manufacturing process. Furthermore, the use of a computer-aided design (CAD) model allows for parallel manufacturing, which in turn facilitates the simultaneous production of multiple AM components, thereby enhancing overall production efficiency. The most common multi-metal additive manufacturing technologies include Directed Energy Deposition (DED), Powder Bed Fusion (PBF), and Binder Jet (BJ). Among these technologies, PBF is distinguished by its ability to produce components with the highest densities and precision, without the need for binder assistance. Furthermore, it ensures high bonding quality at the CuCrZr/SS316L interface and can be applied in the neutron-active region [32].

In this study, we utilized the laser powder bed fusion (LPBF) technique to deposit a 10 mm thick layer of SS316L onto a CuCrZr substrate. Subsequently, we examined the influence of varying laser volumetric energy densities (*E*_v_) on the interfacial hardness of the additive manufacturing (AM) components. To deepen our understanding of the observed changes in interfacial hardness, we correlated scanning electron microscopy (SEM) images with the relevant literature. The quality of interfacial bonding was assessed through tensile experiments conducted under different *E*_v_ conditions. Finally, we present recommended process parameters for the fabrication of dissimilar metal joints, considering interface hardness, microscopic defects, and tensile strength. The objective is to validate the applicability of LPBF technology in producing such joints and to offer guidance for AM technology in the realm of multi-metal structure manufacturing, particularly in the nuclear industry.

## 2. Materials and Methods

### 2.1. Sample Printing Process

The majority of LPBF systems utilize a fiber laser as the energy source, with two output modes: continuous wave (CW) and pulsed wave (PW). The PW output mode is particularly suited to printing multi-metal AM components, due to the laser’s high power density in PW mode coupled with evaporation and recoil effects, which facilitate heterogeneous metal fusion within a smaller heat-affected zone [33,34]. The Renishaw AM400 LPBF system was employed for the fabrication of multi-metal AM components. The CuCrZr substrate was provided in the form of a plate, while the SS316L was supplied as a powder with a particle size range of 10–45 µm. The energy source was a fiber laser with a wavelength of 1070 nm and a spot diameter of 70 µm. The energy output mode is PW, and the programmed adjustments allow for the precise control of scanning power, hatch spacing, spot distance (*D*_p_), and exposure time (*E*_t_). The experiment as a whole included 27 parameter combinations [Table 1]. The laser scanning speeds ranged from 10.36 to 1.6 m/s. After each layer was printed, the laser scanning direction was changed by 90° without any boundary scanning. During the printing of all samples, the thickness of the powder layer was uniformly set to 60 μm, and the hatch spacing was set to 80 μm.

**Figure 2:**
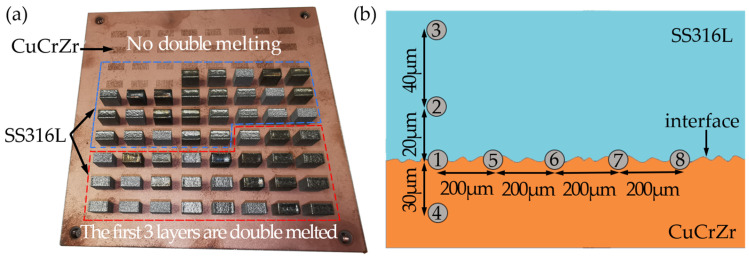
(**a**) Sample experiment; (**b**) schematic diagram of measurement point location.

Two separate methods were used to fabricate the samples due to the potential impact of compositional and thermal property variations on the hardness near the interfaces. The first method involved printing successive layers of powder without remelting each layer, while the second method involved remelting the first three layers as they were printed. To reduce influencing factors and shorten sample fabrication time, all samples were carried out on the same CuCrZr substrate. After the completion of printing, the samples needed to be cut and polished for subsequent measurements. The final product is shown in Figure 2a. The accurate calculation of the volumetric energy density is crucial due to the discontinuous energy output in PW mode, which leads to the discontinuous energy input of the powder layer. The volumetric energy density represents the average energy applied by the laser per unit volume of metal during the scanning process [35] and directly determines the process of the metal melt formation. This, in turn, directly affects the interface hardness measurement results. Thus, it is necessary to correct the volumetric energy density of PW. The laser’s volumetric energy density can be calculated using Equation (1) for CW mode and Equation (2) for PW mode.

(1) $$E_{v}=P/\left(V\times H_{s}\times T\right)$$

(2) $$E_{v}=\delta \times P/\left(V\times H_{s}\times T\right)$$

In this context, the volumetric energy density (*E*_v_) is determined by *D*_p_ and *E*_t_, while *P* represents the scanning power, *V* represents the scanning speed, *H*_s_ represents the hatch spacing, and *T* represents the powder layer thickness. In Equation (2), *δ* represents the duty cycle, which is used as a correction factor ranging from 0.0 to 0.1. According to Brown et al., exposure times of 50 μs, 80 μs, and 110 μs were used for duty cycles of 0.54, 0.75, and 0.90, respectively [36]. Table 1 shows the corrected volumetric energy densities.

### 2.2. Microhardness Test

To prevent small scratches on the sample surface from affecting the results, we polished them after printing [37]. To achieve this, we created a special microhardness test mold using a Schneider Electric PRESSLAM 1.1 hot-mounted press due to the small size of the samples. The samples required stability during the polishing process. The polishing process involved preliminary polishing followed by precision polishing. The preliminary process involved using Struers LaboForce-50 at a speed setting of 300 RPM. We polished the surface until it was free of coarse scratches, followed by a second preliminary process with SiC (220, 500, 1200, 2000, 4000 grits) until the surface was free of visible scratches. Precision grinding was carried out using MD-Largo (DiaPro Largo 9 μm suspension, Struers) until the surface of the sample was free of fine scratches. The samples were then taken for microhardness testing.

Future-Tech FM300e was used for microhardness testing. A constant load of 300 gf was applied during the testing of 54 samples, until there was no further change in the value. The average indentation dwell time was 10 s. Microhardness measurements were taken at selected points on each sample, specifically at points 1, 5, 6, 7, and 8 along the intersection at intervals of 200 μm. Four measurement points were selected perpendicular to the intersection. Points 2 and 3 were located in the SS316L phase, with point 2 at a distance of 20 μm from the intersection, point 3 at a distance of 60 μm, and point 4 at a distance of 30 μm from the intersection in the CuCrZr phase [Figure 2b]. A total of 54 samples were taken following this method. To reduce the possibility of human error, we used the average of the hardness measurements taken at points 1, 5, 6, 7, and 8 to represent the interface hardness. Each point was read by two separate inspectors.

### 2.3. Microhardness Test Uncertainty Analysis Method

**Figure 3:**
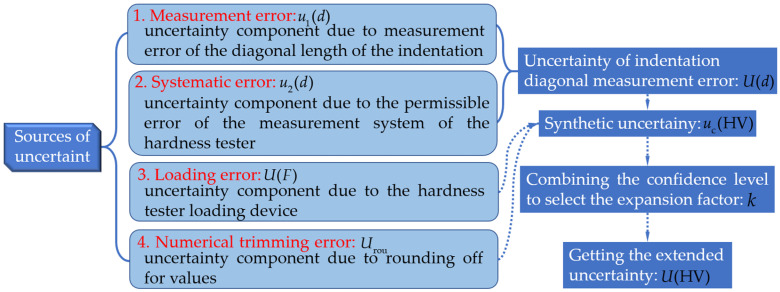
Microhardness test uncertainty analysis process.

To quantify errors in indentation diagonal measurements, hardness tester measurement systems, loading processes, and numerical trimming, an uncertainty assessment is required during microhardness testing [Figure 3]. This analysis process is crucial for ensuring the credibility of the experimental results. Equation (3) is used to calculate the results of material microhardness tests according to the relevant documents published by the American Society for Testing and Materials [38].

(3) $$\mathrm{HV}=\left(2F\sin\frac{136{}^{\circ}}{2}\right)/\left(9.807\times d^{2}\right)=0.1891F/d^{2}$$

In the microhardness measurement, the test force is represented by F, and the arithmetic mean of the diagonal of the two indentations is represented by d. The error in microhardness measurement mainly arises from four aspects [39,40]: (1) the measurement error of the arithmetic mean d of the diagonal of the indentation $u_{1}(d)$; (2) the permissible error of the hardness tester measurement system $u_{2}(d)$; (3) the error of the hardness tester loading device during the loading process U(F); (4) the error introduced by numerical modification $U_{\mathrm{rou}}$. Equations (4)–(7) are used to calculate the error components of the four aspects mentioned above. The uncertainty of the indentation diagonal measurement and the relationship between $u_{1}(d)$ and $u_{1}(d)$ are calculated by Equation (8).

(4) $$u_{1}(d)=\sqrt{\sum_{i=1}^{n}{\left(d_{i}-\bar{d}\right)}^{2}/\left(n^{2}-n\right)}$$

(5) $$u_{2}(d)=a_{1}\times \bar{d}/\sqrt{3}$$

(6) $$U(F)=F\times a_{2}/\sqrt{3}=0.017$$

(7) $$U_{\mathrm{rou}}=0.5\alpha /\sqrt{3}=0.29$$

(8) $$U(d)=\sqrt{u_{1}^{2}(d)+u_{2}^{2}(d)}$$

Among them, $\bar{d}$ is the arithmetic mean of the results of the readings of different measurements at each measurement point; n is the number of measurements; $a_{1}$ is the half-width of the permissible error of the measurement device, according to the standard document, $a_{1}=\pm 1.0\%\bar{d}$; $a_{2}$ is the permissible error of the loading force; in this experiment, the loading force is 300 gf, and the maximum permissible error is ±1.0%F; α is the trimming spacing and numerical trimming of the uncertainty introduced by the uncertainty components in line with the rectangular distribution, α=1. U(F), U(d), $U_{\mathrm{rou}}$ are independent of each other; the synthetic uncertainty is given by the propagation formula (Equation (9)).

(9) $$u_{c}(\mathrm{HV})=\sqrt{c_{1}^{2}U^{2}(d)+c_{2}^{2}U^{2}(F)+c_{3}^{2}U_{\mathrm{rou}}^{2}}$$

where $u_{c}(\mathrm{HV})$ is the synthetic uncertainty; $c_{1}$, $c_{2}$, $c_{3}$ are the sensitivity coefficients, and the specific calculation method and values are shown in Table 2.

The measurement process has an overall confidence level of 95%, and the inclusion factor is *k* = 2. The extended uncertainty is as follows:

(10) $$U(\mathrm{HV})=k\times u_{c}(\mathrm{HV})$$

### 2.4. Tensile Strength Test

**Figure 4:**
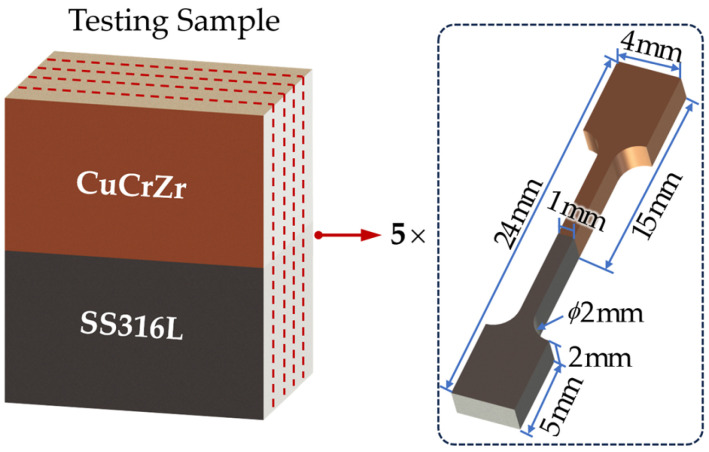
Dimensions of stretched specimen.

To ensure experiment accuracy and minimize errors introduced by sample substitution and specificity, a total of 54 samples were processed for microhardness testing, with the objective of obtaining tensile strength specimens. Initially, the indentations produced during the microhardness test were eliminated by grinding. Subsequently, each sample was divided into five equal portions and subjected to further processing in order to obtain tensile strength specimens (270 in total), as illustrated in Figure 4. The tensile testing equipment used was a Shimadzu Autograph AG-X Plus 10 Kn (Shimadzu, Kyoto, Japan), and the tensile test fixture was attached to the power arm and fixed arm of the tensile testing machine. A 10 mm long gauge was utilized. Data acquisition and processing were performed using a non-contact digital visual tensiometer and its associated software. The instrument boasts an overall accuracy of up to 1.5 μm, thus minimizing equipment-related errors.

Prior to the tensile test, the test equipment and gauges must be calibrated, and the cross-section of each specimen is measured using calipers. During the test, a preload of 10 N was applied, after which the load gradually increased at a constant rate until the specimen broke. We performed tensile tests on all 270 specimens and recorded the results. To minimize errors, the average tensile strength of the five samples from the same print was taken as the tensile strength of the print.

## 3. Results and Discussion

An uncertainty analysis of the key measurement positions is required due to the uncertainties introduced during microhardness testing, such as indentation diagonal measurement errors, loading errors of the hardness tester loading device, numerical trimming, etc. To further reduce uncertainty, the interface hardness is determined as the average value of the hardness of the measurement at points 1, 5, 6, 7, and 8. Table 3 shows the results of the uncertainty analysis for the average hardness at measurement points 1, 5, 6, 7, and 8 under typical conditions.

Upon examining Table 3, it is evident that the uncertainties of the interface hardness measurements for various samples are all within 7%. This suggests that the data are reliable and hold research value.

**Figure 5:**
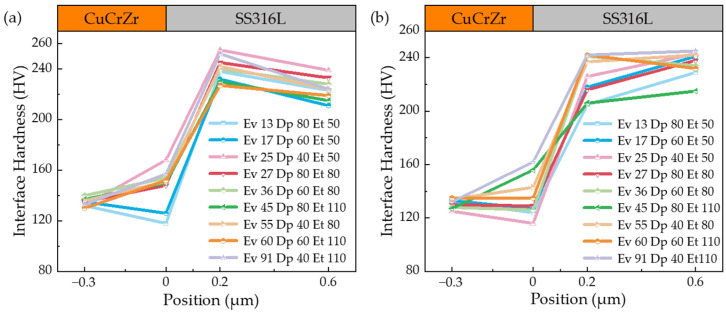
The interface hardness between SS316L and CuCrZr was measured in the transition range using a 200 W laser power. (**a**) Without remelting; (**b**) the first 3 layers are remelted.

**Figure 6:**
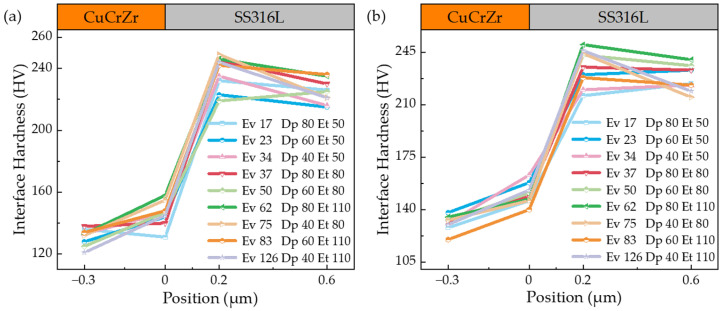
The interface hardness between SS316L and CuCrZr was measured in the transition range using a 275 W laser power. (**a**) Without remelting; (**b**) the first 3 layers are remelted.

**Figure 7:**
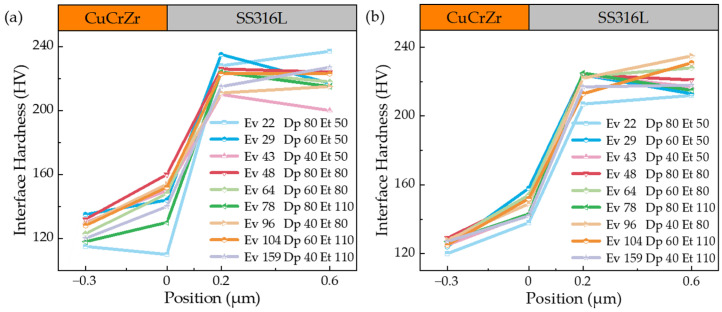
The interface hardness between SS316L and CuCrZr was measured in the transition range using a 350 W laser power. (**a**) Without remelting; (**b**) the first 3 layers are remelted.

**Figure 8:**
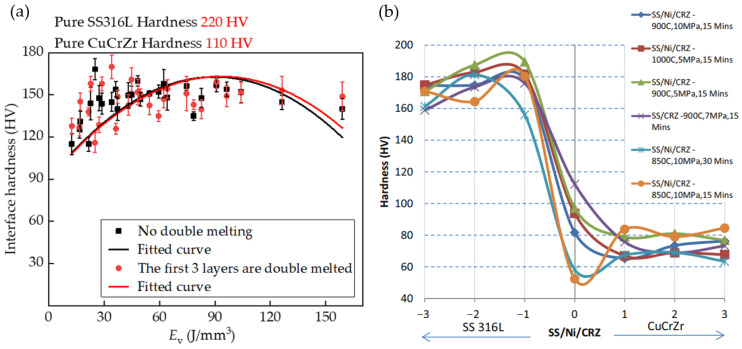
(**a**) The trend of hardness with *E*_v_ observed in SS316L/CuCrZr samples. (**b**) Interfacial microhardness of diffusion-bonded metal joints [10].

Extensive experimental work is required to determine the processing window for the new material combination due to the different laser absorption coefficients and thermal properties inherent in SS316L and CuCrZr. These process parameters are critical for predicting the performance of the experimental samples. Figure 5, Figure 6 and Figure 7 show the hardness profiles of the samples under different parameters, and Figure 8a illustrates the effect of volumetric energy density on interfacial hardness.

**Figure 9:**
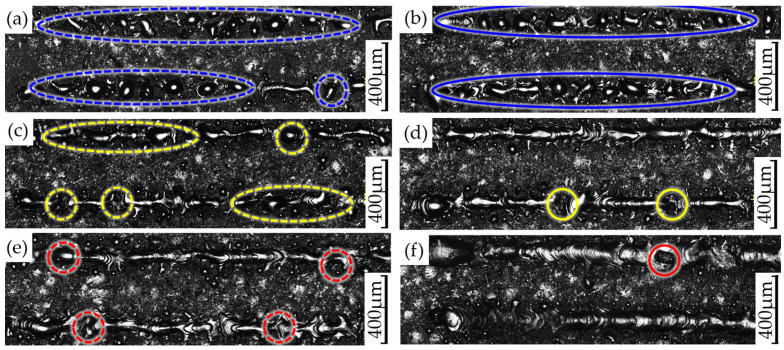
Typical samples in different intervals in the case of no remelting (**left**) and remelting of the first three layers (**right**): (**a**,**b**) *E*_v_ = 13 J/mm^3^, (**c**,**d**) *E*_v_ = 64 J/mm^3^, (**e**,**f**) *E*_v_ = 90 J/mm^3^. Monitoring images of the SS316L/CuCrZr interface captured in the viewing direction parallel to the build and scan directions.

**Figure 10:**
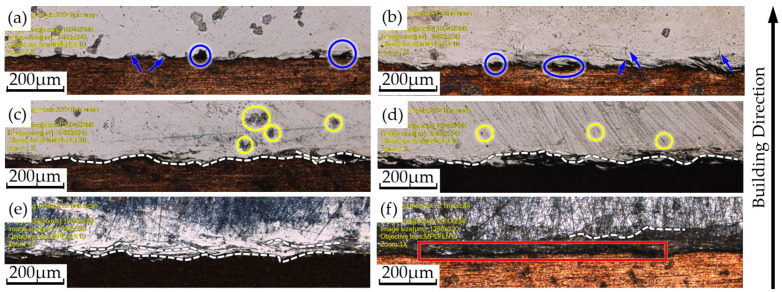
Typical samples in different intervals without remelting (**left**) and with the first three layers remelted: (**right**) (**a**,**b**) *E*_v_ = 13 J/mm^3^, (**c**,**d**) *E*_v_ = 64 J/mm^3^, (**e**,**f**) *E*_v_ = 90 J/mm^3^. SEM images of the SS316L/CuCrZr interface captured in the viewing direction perpendicular to the build and scan directions.

By comparing Figure 5, Figure 6 and Figure 7, it can be seen that the changes in the interfacial hardness of SS316L (hardness 220 HV) and CuCrZr (hardness 110 HV) are similar to the trend of *E*_v_. When *E*_v_ is less than or equal to 17 J/mm^3^, SS316L with a high laser energy absorption coefficient (about 0.5) can be completely melted and allowed to flow sufficiently, and microfluidic forces (surface tension and Marangoni force) can drive the SS316L melt to move. The laser absorption coefficient of CuCrZr is very low (around 0.03), making it difficult to melt. Additionally, as the laser is directly irradiated onto the SS316L powder, some of the energy is absorbed by the SS316L powder, further reducing the energy available for CuCrZr. As a result, CuCrZr only forms a surface layer of melt. At this stage, CuCrZr melts play a lubricating role in the movement of SS316L melt. Microfluidic forces can smoothly drive the SS316L melt along the scanning path relative to the movement of CuCrZr. At this point, the volume of the SS316L melt is small, making it prone to instant solidification into a ball, which prevents the formation of a continuous trajectory [Figure 9a,b]. Due to the poor quality of interphase fusion, when building SS316L, the SS316L side near the interface is prone to holes and cracks, resulting in a clear interface [Figure 10a,b]. When the hardness test probe was pressed on the midpoint of the interface, the high hardness SS316L (220 HV) provides support. However, due to the presence of holes on the SS316L side near the interface, the hardness of the SS316L is lower than that of a standard SS316L (220 HV) member. Therefore, the interface hardness at this point is slightly higher than pure CuCrZr (110 HV), with a hardness range of 110 to 133 HV.

When the laser energy density is between 17 J/mm^3^ and 65 J/mm^3^, the interfacial hardness increases. As the laser energy density increases, SS316L melts sufficiently, and excess energy increases the volume of the CuCrZr melt. This causes the liquid SS316L/CuCrZr ratio volume to decrease, the interphase velocity gradient to decrease, and the microfluidic force to drive the two metal melts to move, forming a relatively continuous trajectory in the construction of SS316L [Figure 9c,d]. The interface between the dissimilar metal phases is relatively clear due to the small size of the melt pool and the extremely short cooling time, which causes the melt to solidify before sufficient diffusion can occur [Figure 10c,d]. When the hardness test probe is pressed on the midpoint of the interface, the SS316L with high hardness (220 HV) provides support, resulting in a hardness range of 135–167 HV. The maximum hardness at the interface is close to the intermediate value between SS316L and CuCrZr (165 HV).

When *E*_v_ > 65 J/mm^3^, the interface hardness decreases. As the laser energy density increases, the CuCrZr melt volume also increases, resulting in a wider print trajectory [Figure 9e,f]. The cooling time increases as the melt pool volume increases due to the effects of microfluidic forces. A larger laser volumetric energy density results in a more intense metal vaporization process, which generates a strong recoil that induces diffusion. The transition between SS316L and CuCrZr is homogeneous, and the interface is gradually blurred [Figure 10e,f]. In this case, the high hardness of SS316L may be diluted by the low hardness of CuCrZr, weakening the supporting role of SS316L and resulting in a decrease in interfacial hardness. The interfacial hardness is now in the range of 132–154 HV, which is reduced compared to the previous stage, along with a slight decrease in overall hardness.

Figure 8b depicts the interfacial microhardness of metal joints achieved through diffusion bonding, as studied by Singh, K. P. et al. [10]. Upon comparison with Figure 5, Figure 6 and Figure 7, it becomes evident that the overall microhardness of metal joints produced by diffusion bonding is lower than that of metal joints obtained through fusion with a laser bed of powders. Additionally, it is essential to incorporate Ni foils between the joints to ensure a robust bond between the dissimilar metals and thereby guarantee the quality of the metal joints in the diffusion bonding process.

To provide a clearer explanation of the impact of *E*_v_ on the interfacial hardness of dissimilar metals, we further compiled and plotted the data from Figure 5, Figure 6 and Figure 7 in Figure 8a. Overall, the interfacial hardness of dissimilar metals gradually increases and then decreases. However, it is important to note that interfacial hardness is not the sole criterion for measuring the quality of parts. Further analysis is needed to address crack defects and internal holes. It is evident that the print trajectory is not continuous when *E*_v_ is less than 17 J/mm^3^ [Figure 9a,b]. The reasons for this are described in detail in the second paragraph of this chapter. Discontinuous trajectories result in holes in the final molded block (shown by blue circles) due to the stacking of these trajectories. This stacking effect is exacerbated by surface tension, resulting in holes with a maximum size of 64.3 μm [Figure 10a]. Microcracks near the interface are present (shown by blue arrows). As per the preceding section, a decrease in *E*_v_ results in a minimal CuCrZr melt, and a fragile copper film with reduced strength forms near the interface during cooling. The difference in thermophysical properties between SS316L and CuCrZr causes their cooling rates to differ, ultimately resulting in high residual stresses in the SS316L phase. This, in turn, leads to the generation of thermal penetration cracks (length 35.7 μm, width 3.2 μm) [Figure 10b]. Therefore, utilizing a lower laser bulk density to manufacture SS316L/CuCrZr samples will lead to relatively low mechanical properties near the interface and overall fatigue life. This situation can be alleviated by adjusting the scanning strategy and reducing the print speed, but it will significantly increase the print time. When the energy density is between 17 J/mm^3^ and 65 J/mm^3^, the melt pool continuity improves significantly. However, small gaps may still form (indicated by the yellow dotted circle) [Figure 9c,d], with a maximum size of 48 μm. These gaps can be reduced by adjusting the scanning spacing and ensuring good overlap, taking into account surface tension and molecular forces. The size of the hole is also small (maximum size 35.3 μm) [Figure 10c]. There is no visible crack. This is because an increase in *E*_v_ leads to an increase in the melt pool volume and cooling time, as well as an increase in the degree of the alloying of dissimilar metals. Although residual stresses are present, they are relatively evenly distributed and not sufficient to cause visible cracks. When the laser volumetric energy density exceeds 65 J/mm^3^, the metal powder’s molten pool may be blown away by the large vaporization recoil during the printing process [41], resulting in regularly shaped pores (maximum size 84 μm) [Figure 9e]. However, we observed that no significant pores were formed in the final block. This is because, at higher *E*_v_ values, the molten pool can easily remelt the already solidified pool of the previous layer and fill up the pores that existed in the previous layer. Nevertheless, the interfacial hardness in the range is relatively low due to the high degree of alloying.

When comparing the Marangoni force-affected region (shown by the white dashed line) in Figure 10 vertically, it is clear that the wavy interface that formed due to the Marangoni force is not as prominent in the lower *E*_v_ case (≤17 J/mm^3^) [Figure 10a,b]. As *E*_v_ increases (17 J/mm^3^ < *E*_v_ ≤ 65 J/mm^3^), the Marangoni force becomes increasingly prominent. This results in a non-uniform, wavy interface [Figure 10c,d]. The interface exhibits curvature, and there are signs of the intrusion of CuCrZr into the SS316L phase, indicating a tendency towards the formation of a transition zone. As *E*_v_ continues to increase (65 J/mm^3^ < *E*_v_), the wavy interface is evident and uniform, and a clear transition zone has formed (red box) [Figure 10e,f], suggesting that the Marangoni forces have influenced the time to increase the alloying degree of SS316L/CuCrZr, which also corroborates the previous arguments. A comparison of the SS316L/CuCrZr images captured perpendicular and parallel to the scanning and building directions in both printing modes reveals that defects such as cracks and holes can be improved in samples built by remelting, regardless of the *E*_v_ interval. Ge, Feiyu, Lyu, Peng, Xiong, and Zhengang et al. [42,43,44] suggest that remelting can refine grains. During the cooling of the prints, the coarse grains tend to produce large internal stresses, resulting in stress concentration areas at grain boundaries, which increases the possibility of cracks and holes appearing. Smaller grains experience lower internal stresses during the cooling process, reducing the likelihood of cracks and holes forming at the grain boundaries.

**Figure 11:**
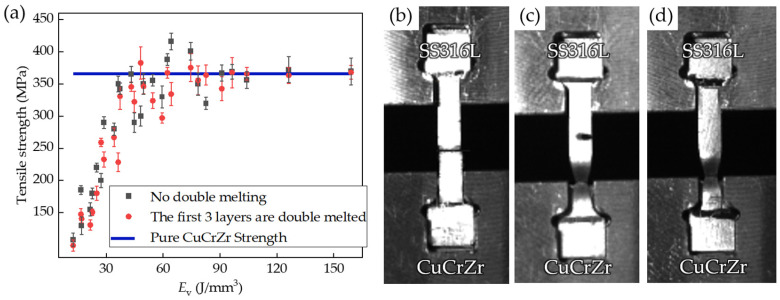
(**a**) Trend of tensile strength with *E*_V_ for different SS316L/CuCrZr samples; (**b**) *E*_v_ = 13 J/mm^3^ and (**c**) *E*_v_ = 54 J/mm^3^; (**d**) fracture position at *E*_v_ = 90 J/mm^3^.

To further verify the feasibility of using additive manufacturing technology to produce metal joints, tensile strength tests are also required. We plotted the tensile strength curves at different *E*_v_ based on the tensile test measurements [Figure 11a]. It is evident that the tensile strength gradually increases with the increase in *E*_v_, and the tensile strength curve stabilizes around 360 MPa after *E*_v_ exceeds 60 J/mm^3^. Additionally, it is observed that the tensile strength curve in the remelted case exhibits better consistency after surpassing 60 J/mm^3^ compared to the curve without remelting. Combining these observations with the previous analysis, when *E*_v_ ≤ 20 J/mm^3^, the tensile strength is only 108–147 MPa. This is attributed to the minimal alloying between dissimilar metals and the presence of holes and microcracks in the tensile specimens, resulting in significantly lower tensile strength compared to SS316L and CuCrZr. At this stage, the fracture tends to occur near the interface, displaying characteristics of brittle fracture [Figure 11b]. When 20 J/mm^3^ < *E*_v_ ≤ 60 J/mm^3^, the tensile strength is in the range of 135–358 MPa. This improvement is attributed to the increased degree of alloying at the interface, reduced presence of voids and microcracks, and enhanced mechanical properties with the increase in *E*_v_. Despite the fracture surface still being near the interface, clear ductility is observed on both sides of the fracture surface [Figure 11c]. When 60 J/mm^3^ < *E*_v_, there is still noticeable ductility near the interface, but the fracture surface may occur on the CuCrZr side, which is because the increase in *E*_v_ significantly enhances the alloying degree of the interface, resulting in the tensile strength of the interface metal near or even exceeding that of CuCrZr [Figure 11d]. In addition, since remelting aids in promoting fusion between dissimilar metals and addresses issues such as holes and microcracks, the samples produced by remelting exhibit better consistency, i.e., more stable mechanical properties, in the tensile strength images.

Considering the differences in hardness at the interface of dissimilar metals, surface defects and internal holes, and tensile strength, it is recommended to choose 60 J/mm^3^ < *E*_v_ < 90 J/mm^3^, where the microgap and energy consumption are relatively low, the average interface hardness is about 150 HV, and the tensile strength is about 360 MPa. Furthermore, it is advised to utilize remelting to construct the block adjacent to the dissimilar metal interface, which can further enhance microscopic defects such as holes and cracks at the dissimilar metal interface, thereby rendering the mechanical properties more stable.

## 4. Conclusions

In this study, we employed the LPBF technique to deposit 10 mm thick SS316L on CuCrZr. We then investigated the effect of different laser volumetric energy densities (*E*_v_) on the interfacial hardness of the AM component (SS316L/CuCrZr). Additionally, we analyzed the effect of cracks, pore defects near the interface, and the effect of the alloying degree on the interfacial hardness. As the energy density increases, the melt pool gradually expands, the cooling time increases, the degree of alloying at the interface increases due to the Marangoni force, and the interface hardness increases and then decreases. The interface hardness is highest at 65 J/mm^3^ ≥ *E*_v_ > 17 J/mm^3^ (135~167 HV), and the interface hardness after the decrease is still close to the midpoint of the hardness of SS316L and CuCrZr (165 HV). In order to further verify the feasibility of LPBF technology to produce SS316L/CuCrZr joints, tensile experiments were conducted to evaluate the interfacial bonding quality of different samples. The tensile strength of the specimens was found to be approximately 360 MPa when the *E*_v_ exceeded 60 J/mm^3^, which was already close to that of the lower tensile strength base material (CuCrZr). Regarding the interface hardness and tensile strength, the recommended *E*_v_ range is 60 to 90 J/mm^3^, with an average interface hardness of approximately 150 HV and satisfactory bonding quality. In comparison to the SS316L/CuCrZr metal joints produced by the HIP diffusion joining technique, the interfacial hardness exhibited an increase of 30% to 40%, while the CuCrZr hardness degradation was significantly improved. These studies show that LPBF technology can be applied to the production of SS316L/CuCrZr joints in the field of nuclear industry in terms of microhardness and tensile strength, and it can be used as a reference for multi-metal material problems such as the reinforced repair of radiators and pipes in the same field.

## Figures and Tables

**Table 1:** Process parameters of LPBF.

*P* (W)	*D*_p_ (μm)	*E*_t_ (μs)	*V* (m/s)	*E*_v_ (J/mm^3^)
200	80	110	0.73	45
		80	1.00	27
		50	1.60	13
	60	110	0.55	60
		80	0.75	36
		50	1.20	17
	40	110	0.36	91
		80	0.50	55
		50	0.80	25
275	80	110	0.73	62
		80	1.00	37
		50	1.60	17
	60	110	0.55	83
		80	0.75	50
		50	1.20	23
	40	110	0.36	126
		80	0.50	75
		50	0.80	34
350	80	110	0.73	78
		80	1.00	48
		50	1.60	22
	60	110	0.55	104
		80	0.75	64
		50	1.20	29
	40	110	0.36	159
		80	0.50	96
		50	0.80	43

**Table 2:** Methods or values for calculating sensitivity factors.

Sensitivity Factors	Methods or Values
$c_{1}$	$\partial \mathrm{HV}/\partial d=\left(-2\times 0.1891\times F\right)/{\bar{d}}^{3}$
$c_{2}$	$\partial \mathrm{HV}/\partial F=0.1891/{\bar{d}}^{2}$
$c_{3}$	1

**Table 3:** Measurement uncertainty of interfacial hardness with different parameters.

Manufacturing Method	Process Parameters	HV	Uncertainty Calculation			
			$\bar{d}$(μm)	U(d)(μm)	$u_{c}(\mathrm{HV})$	U(HV)
Without remelting	*P* = 200 W *D*_p_ = 80 μm *E*_t_ = 50 μs	118	68.664	1.113	3.943	7.885
	*P* = 275 W *D*_p_ = 80 μm *E*_t_ = 50μs	131	65.168	0.89	3.665	7.329
	*P* = 275 W *D*_p_ = 40 μm *E*_t_ = 50 μs	147	61.512	0.68	3.355	6.711
	*P* = 275 W *D*_p_ = 40 μm *E*_t_ = 80 μs	155	59.91	0.93	4.889	9.779
	*P* = 350 W *D*_p_ = 40 μm *E*_t_ = 110 μs	146	61.729	0.76	3.7	7.4
The first 3 layers are remelted	*P* = 200 W *D*_p_ = 80 μm *E*_t_ = 50 μs	124	66.981	1.14	4.289	8.577
	*P* = 275 W *D*_p_ = 80 μm *E*_t_ = 50 μs	146	61.729	1.01	4.874	9.747
	*P* = 275 W *D*_p_ = 40 μm *E*_t_ = 50 μs	163	58.422	0.97	5.511	11.023
	*P* = 275 W *D*_p_ = 40 μm *E*_t_ = 80 μs	146	61.729	0.83	4.024	8.048
	*P* = 350 W *D*_p_ = 40 μm *E*_t_ = 110 μs	142	62.593	1.04	4.803	9.607

## Data Availability

Data are contained within the article.

## Abbreviations

HIP	Hot isostatic pressing
LPBF	Laser powder bed fusion
SEM	Scanning electron microscopy
AM	Additive manufacturing
CW	Continuous wave
PW	Pulsed wave
Symbols and units
Scanning power: *P*	W
Spot distance: *D*_p_	μm
Hatch spacing: *H*_s_	μm
Powder layer thickness: *T*	μm
Exposure time: *E*_t_	μs
Scanning speed: *V*	m/s
Volumetric energy density: *E*_v_	J/mm^3^
Duty cycle: *δ*	dimensionless
Uncertainty of indentation diagonal measurement error: U(d)	mm
Measurement error: $u_{1}(d)$	mm
Systematic error: $u_{2}(d)$	mm
Loading error: U(F)	N
Numerical trimming error: $U_{\mathrm{rou}}$	N/mm^2^
Synthetic uncertainty: $u_{c}(\mathrm{HV})$	N/mm^2^
Extended uncertainty: U(HV)	N/mm^2^
